# Impact of *Washingtonia robusta* Leaves on Gamma Irradiation-Induced Hepatotoxicity in Rats and Correlation with STING Pathway and Phenolic Composition

**DOI:** 10.3390/ph13100320

**Published:** 2020-10-19

**Authors:** Nabil M. Selim, Seham S. El-Hawary, Soheir M. El Zalabani, Rehab Nabil Shamma, Nariman El Sayed Mahdy, Noheir H. Sherif, Hanan A. Fahmy, Mai H. Mekkawy, Abdelaziz Yasri, Mansour Sobeh

**Affiliations:** 1 Department of Pharmacognosy, Faculty of Pharmacy, Cairo University, Cairo 12613, Egypt; seham.elhawary@yahoo.com (S.S.E.-H.); selzalabani@gmail.com (S.M.E.Z.); narimanmahdy@yahoo.com (N.E.S.M.); 2 Department of Pharmaceutics and Industrial Pharmacy, Faculty of Pharmacy, Cairo University, Cairo 12613, Egypt; rehab.shamma@pharma.cu.edu.eg; 3 Pharmacognosy Department, Faculty of Pharmacy, Nahda University, Beni-Suef 62513, Egypt; noheir_1@hotmail.com; 4 Drug Radiation Research Department, National Centre for Radiation Research and Technology, Egyptian Atomic Energy Authority, P.O. Box: 29 Nasr City, Cairo 11865, Egypt; fahmy.hanan@yahoo.com (H.A.F.); maimekkawy@hotmail.com (M.H.M.); 5 AgroBioSciences Research Division, Mohammed VI Polytechnic University, Lot 660–Hay MoulayRachid, 43150 Ben-Guerir, Morocco; aziz.yasri@um6p.ma

**Keywords:** *Washingtonia robusta*, *Washingtonia filifera*, antioxidant, phenolics, γ-irradiation, hepatoprotective, STING gene expression

## Abstract

Exposure to ionizing radiation usually results in cellular oxidative damage and may induce liver toxicity. The efficiency of the ethanol extracts of *Washingtonia filifera* (EWF) and *Washingtonia robusta* (EWR) leaves in alleviating γ-radiation-induced oxidative hepatotoxicity was herein explored. Proximate and macronutrient composition of the leaves was determined to establish reliable quality control criteria. Colorimetric estimation of total phenolic (TPC) and flavonoid (TFC) contents revealed their occurrence in larger amounts in EWR. In vitro evaluation of the antioxidant capacity by 2,2-azinobis (3-ethylbenzothiazoline-6-sulfonic acid (ABTS) and ferric reducing antioxidant power (FRAP) assays confirmed higher efficiency of EWR designating a close correlation with phenolic composition. Four phenolics, viz., naringenin, kaempferol, quercetin, and gallic acid, were isolated from EWR. In vivo assessment of the extracts’ antioxidant potential was performed on γ-irradiated (7.5 Gy) female rats. EWR was found more efficient in restoring the elevated liver index, ALT, albumin, cholesterol, and reactive oxygen species (ROS) levels. Both extracts ameliorated the increase in the stimulator of interferon gene (STING) expression. Bioactivity was confirmed by immuno-histochemical examination of inflammatory and apoptotic biomarkers (TNF-α, IL-6 and caspase-3) and histopathological architecture. In addition, the interactions of the isolated compounds with STING were assessed in silico by molecular docking. Therefore, *Washingtonia robusta* leaves might be suggested as a valuable nutritional supplement to alleviate radiotherapy-induced hepatotoxicity.

## 1. Introduction

Radiotherapy is one of the most common methods that are partially used in the treatment and control of cancer. Exposure to ionizing radiation induces oxidative stress, mainly via production of reactive oxygen species (ROS) that cause deleterious damage to different cellular macromolecules and enhance the pathogenesis of various disorders including liver injury [1]. These outcomes have triggered experts to search for potent and safe remedies from natural sources that could mitigate the damage caused by radiotherapy.

STING (cGAS-stimulator of interferon genes) is found in the endoplasmic reticulum and expressed in immune and non-immune cells [2]. Radiation induces DNA damage through generation of ROS, then DNA translocate to cytosol. Cytosolic DNA stimulates the cGAS-cGAMP-STING signaling [3]. Activated STING activates the nuclear factor kappa B (NF-ĸB) pathway through the tumor necrosis factor (TNF) receptor [4]. The activated NF-ĸB enters the nucleus and increases the expression of inflammatory cytokines such as TNF-α and interlukin-6 (IL-6) [5]. Stimulation of the STING pathway leads to inflammation and cell metabolic conditions such as disturbance in fat metabolism and glycolysis [6]. Furthermore, STING activation in certain cell types elicited cell death including apoptosis and necrosis [7].

Arecaceae (Palmae or palm family) is a monophyletic group consisting of about 200 genera and 2700 species [8] with geographical distribution almost restricted to tropical and subtropical regions. The family comprises five subfamilies including Coryphoideae. The latter is further subdivided into eight tribes; among these is tribe Trachycarpeae to which belongs genus *Washingtonia* H. Wendl. alongside eighteen other genera. Two species are recognized in genus *Washingtonia*, namely *Washingtonia filifera* H. Wendl. (known as the California fan palm) and *Washingtonia robusta* H. Wendl. (or Mexican fan palm) [9], and successfully acclimatized in Egypt. The economic importance of members of this family is owed to their edible fruits as coconuts, areca nuts, and date palms, as well as their anti-oxidant and anti-cancer potentials; in addition, a large number are cultivated as ornamentals [10,11]^.^

Most Trachycarpeae plants are characterized by the prevalence of lipoids (triterpenoids and steroids), polyphenols (flavonoids and phenolic acids), and saponins, as major bioactive secondary metabolites [12,13,14]. Moreover, several scientifically-based bioactivities are ascribed to members of this tribe including anti-inflammatory, antioxidant, antiprotozoal, antidiabetic, anti-hyperlipidemic, antimicrobial, antiviral, anti-benign prostate hyperplasia, and anti-prostatic cancer [14,15,16,17].

Chemical investigation of the aerial parts of *W. filifera* resulted in isolation of two new flavonoids, 8-hydroxyisoscoparin, and luteolin 7-*O*-glucoside 4″-sulfate, along with eight other known flavones [18]. Later on, a heterocyclic bioactive quinazoline-2, 4-(1*H*, 3*H*) dione was isolated from the leaves of the plant [19]. Recently, a chalconoid analogue, 1,3,5-benzentriol 2-[(2*S*,3*R*)-3-(3,4-dihydroxylphenyl)-2,3-dihydroxylpropyl], known as filiferol, was reported from the leaves [20]. Meanwhile, data on scientifically based bioactivities of *W. filifera* were relatively scarce and restricted to in vitro evaluation of its antioxidant and antifungal effects [18,19,20], whereas, limited reports could be traced concerning either the chemical composition or biological activity of *W. robusta*.

In the current study, the antioxidant capacities of the ethanol extracts of *W. filifera* and *W. robusta* leaves were assessed in vitro by 2,2-azinobis (3-ethylbenzothiazoline-6-sulfonic acid (ABTS) and ferric reducing antioxidant power (FRAP) assays, and correlated to their spectrophotometrically determined phenolic contents. This was in view of further in vivo evaluation of their respective potentialities in enhancing liver recovery processes in γ-irradiated rats. Prior to chemical and biological investigation, the tested samples were characterized through proximate and macronutrient analyses.

## 2. Results and Discussion

### 2.1. Chemical Examination and In Vitro Antioxidant Potential of the Leaves

To ensure purity and detect adulteration or contamination, quality control criteria were established for the leaves of the two species through proximate and macronutrient analyses. These included determination of total ash (1.37 and 1.64 g/100 g), acid-insoluble ash (0.31 and 0.35 g/100 g), water-soluble ash (0.88 and 1.15 g/100 g) values, in addition to crude fiber (10.65 and 15.78 g/100 g) and moisture (48.25 and 50.85 g/100 g) contents. The amount of crude fibers in *W. robusta* leaves exceeded by far that in those of *W. filifera,* pointing to a greater content of indigestible polysaccharides and lignin. On the other hand, the lipid and carbohydrate contents and total energy value of *W. filifera* sample were higher than in its *W. robusta* analogue (1.47 vs. 1.17 g/100 g, 27.91 vs. 19.17 g/100 g, and 166.27 vs. 132.77 Kcal /100 g, respectively); in contrast, its protein content was slightly lower (10.35 vs. 11.39 g/100 g in *W. robusta*). The relatively high moisture, fiber, and protein contents, besides the considerable amount of carbohydrates and low-fat content recorded in the leaves of the two species, support their use in controlled calorie diets with optimal nutritional value.

A qualitative similarity in metabolic composition was observed in the aforementioned extracts. Both gave a strong positive response to tests for carbohydrates and/or glycosides, free and combined flavonoids, sterols, and/or triterpenes, and a weaker one to those of anthraquinones, tannins, and saponins. The organoleptic characters of the leaves extractives were almost matching, yet the yield of those of *W. robusta* were slightly higher (Table 1).

Spectrophotometric determination of total phenolic and flavonoid contents (TPC and TFC) in the two leaves samples revealed that TPC of the ethanol extract of *W. robusta* (EWR) was much higher (by about 2.5 times) than that of *W. filifera* (EWF) (reaching 215.51 ± 0.28 vs. 79.58 ± 0.24 μg GAE/mg extract). Likewise, TFC of EWR was slightly higher than its EWF analogue (64.40 ± 0.14 vs. 54.68 ± 0.46 μg QE/mg extract).

Reports concerning the antioxidant activity of *W. filifera* leaves are quite few and confined to its assessment using DPPH (2,2-diphenyl-1-picrylhydrazyl) [19] and β-carotene bleaching [18] procedures. Meanwhile, nothing could be traced concerning that of *W. robusta.* Alternative in vitro methods, viz., ABTS and FRAP assays [21,22,23,24,25,26], were, herein, adopted for evaluation of the antioxidant potential of the leaves of the two species. Results (Table 2) indicated that both samples exert significant antioxidant potential in comparison to the reference drug, ascorbic acid. Yet, those of *W. robusta* were found more effective; this was in accordance with their wealthier phenolic content, and implied deeper investigation of their bioactive components.

**Figure 1:**
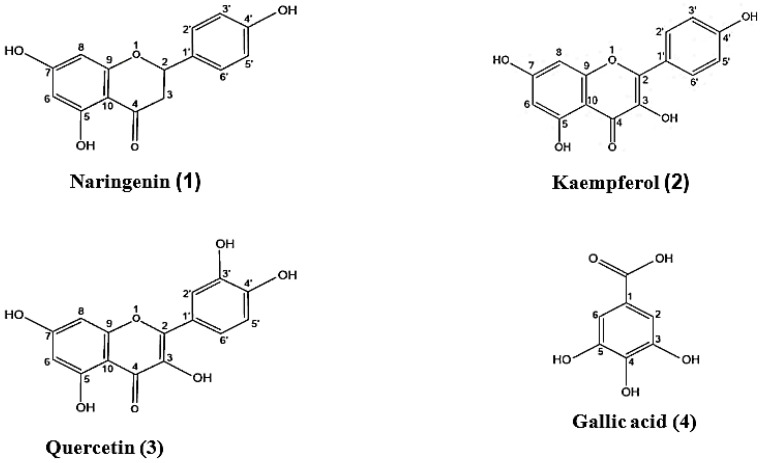
Compounds isolated from the ethyl acetate extract of *W. robusta* leaves.

Four known phenolic compounds, a flavanone (naringenin), two flavonols (kaempferol and quercetin), and a phenolic acid, (gallic acid) (Figure 1), were isolated from the ethyl acetate fraction of the ethanol extract of *W. robusta* (EWR) through successive solvent extraction followed by repeated column fractionation and purification. The isolated secondary metabolites were characterized via physicochemical examination, spectral analysis (UV, ^1^H NMR, and ^13^C NMR), and by correlation to former reports [27,28,29,30,31]. Despite being widely distributed in nature, this is the earliest information concerning the isolation of these compounds from the leaves of *W. robusta*. Yet, quercetin and naringenin have been formerly identified in certain members of tribe Trachycarpeae, namely *Brahea armata* S. Watson and *Chamaerops humilis* L. [32,33]. UPLC/MS (ultra performance liquid chromatography coupled with mass spectroscopy) metabolic profiling of the ethanol extract of the leaves of *W. robusta* has been performed. This allowed the tentative identification of 60 compounds including 3,4-dimethoxy-dihyro cinnamic acid methyl ester, catechin, epicatechin, isorhamnetin-3-glucoside-4′-glucoside, protocatechuic acid, among others, which may contribute synergistically to the bioactivity of the compounds isolated herein (data are not shown).

### 2.2. In Vivo Median Lethal Dose (LD_50_) Determination

The acute toxicity study of the leaves of *W. filifera* and *W. robusta* was carried out in vivo in albino mice, and the median lethal doses (LD_50_) were determined and found to be 2500 and 3500 mg/kg b.wt., (i.p.), respectively. Thus, both EWF and EWR could be considered as safe for administration to the experimental animals. Yet, EWR appeared to be safer due to its higher LD_50_. Two doses (100 and 300 mg/kg b.wt.) of each extract were then chosen, as therapeutic doses, for subsequent estimation of its antioxidant activity and modulatory effect toward radiation-induced oxidative stress and liver damage.

### 2.3. Effect on Oxidative Stress Parameters

The role of ionizing radiation in increasing the production of reactive oxygen species (ROS), such as O_2_^•−^, OH^•^ and organic hydroperoxides in a variety of cells has been proved [34,35]. These ROS cause DNA strand break, lipid peroxidation (LPO), and protein modification [36]. The antioxidant effect of the tested extracts was evaluated through determination of oxidative stress parameters in blood samples of irradiation-liver injured rats including malondialdehyde (MDA) and ROS, as represented in Table 3.

The recorded data (Table 3) revealed that treatment of rats with EWF or EWR at both doses exhibited non-significant changes in the MDA and ROS levels compared to control group, reflecting a safe use of these extracts, except for F300, which caused an elevation of ROS levels. Exposure of rats to γ-radiation resulted in a significant increase by 40.7% and 75.5% in the levels of MDA and ROS in comparison to control values (*p* ≤ 0.05). Elevation in the oxidative stress could be due to the generation of metabolites that rise the body burden of free radicals, as well as ROS generation from water radiolysis [34,35].

Treatment of irradiated rats with EWF or EWR at both doses exhibited a significant reduction in MDA and ROS levels until close to the control values, except for FIR100, which failed to modulate the ROS levels. The marked reduction in ROS and lipid peroxides levels in rats post-treated with the ethanolic extracts of the two leaves, especially EWR, could be due to inhibition of the peroxidation process through scavenging free radicals (decreasing ROS content) and thus alleviating the harmful effects of oxidative stress of gamma radiation.

### 2.4. In Vivo Genetic Profiling of the Stimulator of Interferon Gene (STING) and Molecular Docking of the Isolated Compounds

**Figure 2:**
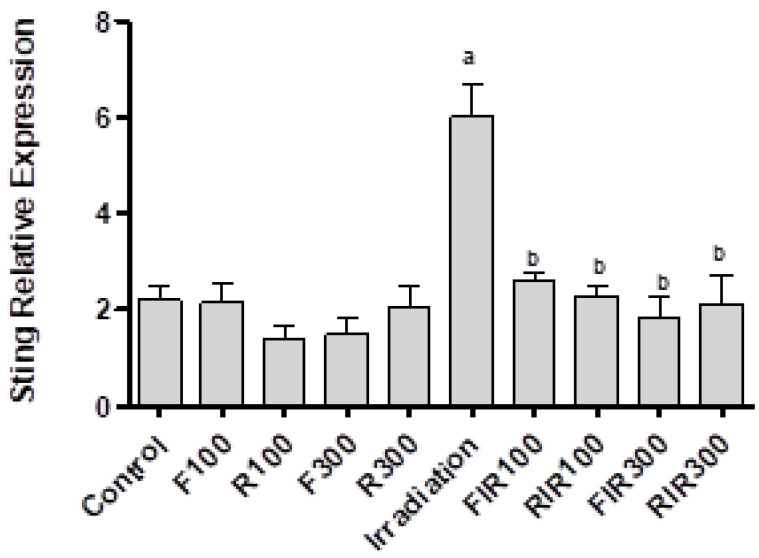
Effect of *W. filifera* and *W. robusta* leaves ethanolic extracts on hepatic stimulator of interferon gene (STING) relative expression in irradiated rats. Data are expressed as mean ± SD, significance was at *p* ≤ 0.05 by means of one-way ANOVA followed by Tukey–Kramer as a post hoc-test. ^a^ Significantly different as of control group. ^b^ Significantly different as of irradiated group.

Stimulation of STING gene activates an inflammatory reaction due to the presence of cytosolic DNA [37]. Therefore, the search for natural products that inhibit STING provides important insight therapies to treat inflammatory disease. Real-time PCR showed that the STING gene was overactivated by exposing rats to γ-radiation and became significantly changed compared to the normal control group (Figure 2). Activation of the STING gene may be due to the damage of mitochondrial DNA (mtDNA) caused by ionizing radiation, resulted in its cytosolic translocation and activation of the cytosolic STING [38,39].

A significant diminution in the expression of the STING level was recorded in the groups exposed to γ-radiation and receiving two different doses of EWF or EWR as compared to the γ-irradiated group due to inhibition of the activation process. Non-irradiated rats treated with both extracts displayed insignificant changes as compared to the normal control group, confirming their safety. Our study on gene expression in liver cells revealed that nearly the same results were obtained for both extracts on STING levels against γ-irradiation. An increase in the endogenous antioxidant level by EWF and EWR may be responsible for the scavenging of radiation-induced free radicals, thus protecting against radiation-induced STING activation.

**Figure 3:**
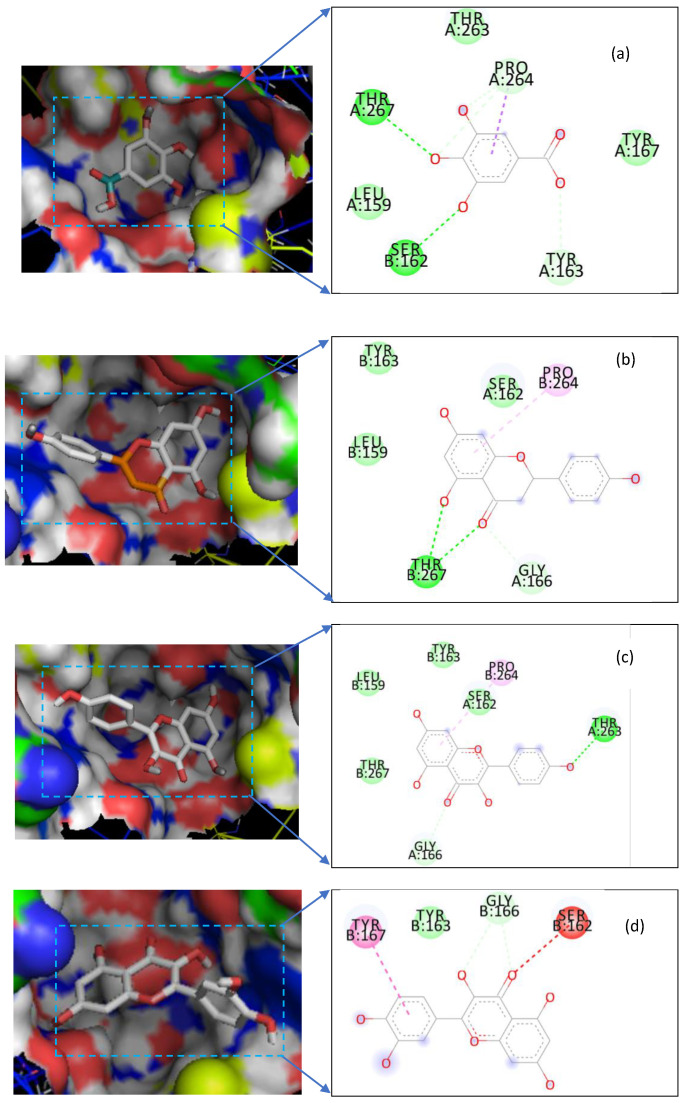
Compounds’ binding mode to STING receptor and interaction maps. (**a**) Gallic acid; (**b**) Naringenin; (**c**) Kaempferol; (**d**) Quercetin.

Most of the compounds docked within the STING receptor gave binding energy values between −5.7 Kcal/Mol (gallic acid) and 7.2 kcal/Mol (naringenin). When we analyzed the binding modes of the compounds to the receptor (Figure 3), we found that 6 amino acids (Leu-159, Ser-162, Tyr-163, Gly-166, Thr-263, and Pro-264) were the most concerned by these interactions which are of the Van der Wals, hydrogen bonding, and Pi-sigma interactions type. The elucidation of these interactions and of the modes of binding of these compounds open up new avenues for medicinal chemists to optimize new agonists of the STING receptor or to design new ones.

### 2.5. Effect on Liver Index, Hepatic Biomarkers, and Lipid Profile

**Figure 4:**
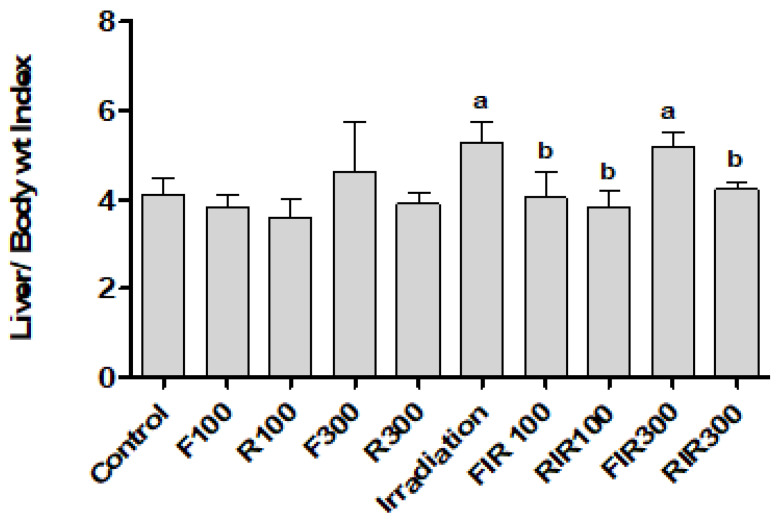
Effect of *W. filifera* and *W. robusta* leaves ethanolic extracts on liver index in irradiated rats. Data are expressed as mean ± SD, significance was at *p* ≤ 0.05 by means of one-way ANOVA followed by Tukey–Kramer as a post hoc-test. ^a^ Significantly different as of control group, ^b^ significantly different as of irradiated group (*n* = 6).

Whole body gamma-irradiation may result in changes in liver structure and incidence of liver damage. It was reported that the increase of liver weight after radiation exposure can be attributed to the accumulation of glycogen in liver post irradiation [40]. In correlation to control values (*p* ≤ 0.05), non-significant changes in liver/body weight ratio were recorded in animals treated with tested extracts alone, without irradiation, confirming their safety, while a significant increase of 28.2% was recorded when the animals were exposed to γ-radiation. Administration (i.p.) of FIR100 or RIR at both doses for 7 days after gamma exposure significantly declined the liver weight ratio as compared to irradiated group. However, data showed that FIR300 failed to restore relative liver weight in rats exposed to γ-radiation (Figure 4).

Concerning the radiation effect on liver biomarkers, rats exposed to gamma radiation (7.5 Gy) reveal an increase in the serum alanine transaminase (ALT) (75%) activity, cholesterol (145%) concentration, and triglyceride concentration (TG) (50%), along with a significant decrease in the albumin (32.4%) concentration in comparison with those of control values. These results are in accordance with those of Mekkawy et al. 2020 and Sayed et al. 2020 [41,42]. They reported that γ–irradiation-induced oxidative stress elevates the permeability of cell membranes and enables the leakage of enzymes outside the cells. In addition, the hyperlipidemic state caused by gamma irradiation could be attributed to the stimulation of cholesterol synthesis by promoting the coenzyme A (HMG-CoA) reductase activity which stimulates cholesterol synthesis [42]. In addition, as established previously in this study, gamma radiation activates STING, and activation of the STING-interferon 3 pathway (STING-IRF3) triggers hepatocyte damage and dysfunction by increasing IL-6 and TNF-α, thus eliciting inflammation and apoptosis that disturb lipid metabolism [6]. Additionally, the elevation in IL-6 caused by gamma radiation reduces the production of albumin and transferrin [43]. Changes in ALT, albumin, cholesterol, and triglyceride levels were observed in the serum of rats upon treatment with ethanolic leaf extracts of the two plants, as indicated in Table 4. The results showed, as well, insignificant changes in serum ALT levels of rats treated with EWF or EWR at both doses as compared to the normal control level, which assured their safety. Administration of EWF (100 mg/kg) after γ-irradiation failed to improve the ALT activity. However, rats exposed to γ-irradiation then treated with both doses of EWR and EWF (300 mg/kg) showed a significant decrease in serum ALT as compared to irradiated group. Whereas animals treated with EWR after exposure to γ-radiation restored the albumin levels nearly to control values. However, EWF failed to renormalize the albumin values compared to its normal control levels. Groups of rats exposed to γ-radiation and treated with both doses of EWR produced a significant restoration in cholesterol levels; the data recorded are more or less similar to control values. However, rat irradiation (7.5 Gy) followed by EWF administration (i.p.) showed partial improvement in the recorded level relative to EWR. The current data revealed that EWR at both doses could mitigate the harmful effects of gamma radiation-induced liver damage by decreasing the elevated triglyceride, cholesterol, and ALT levels and increasing the albumin level; this could be attributed to its hepatoprotective effect. This extract might also hinder the detrimental effects of free radicals by preventing the oxidation of cell membrane.

### 2.6. Histopathological Examination

**Figure 5:**
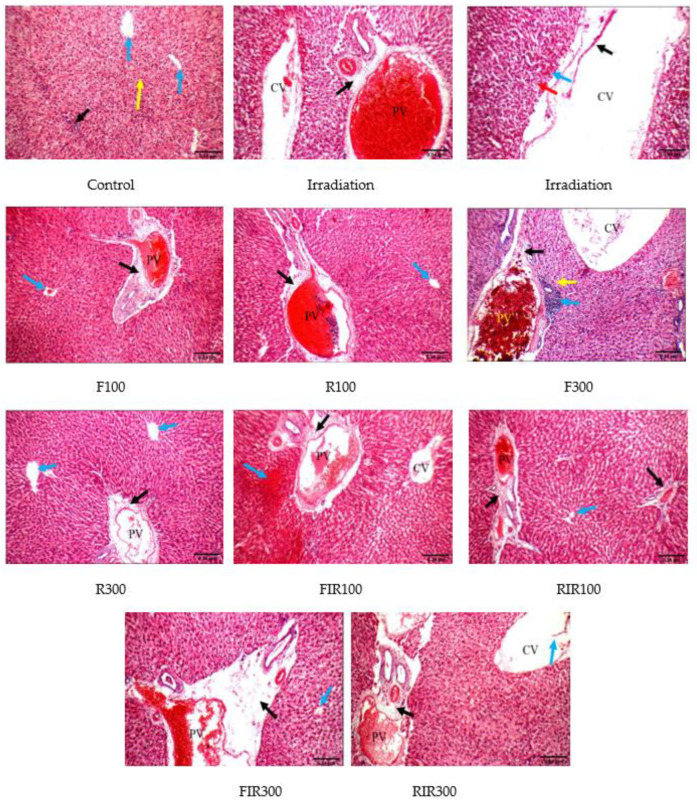
Histopathological examination of liver in irradiated rats treated with *W. filifera* and *W. robusta* leaves ethanolic extract (H and E; ×200). Control: Liver specifying average portal tract (black arrow), average central veins (blue arrows), and average hepatocytes (yellow arrow). IR: Liver showing markedly edematous portal tract (black arrow) with markedly dilated congested portal vein (PV) and markedly dilated congested central vein (CV); another view showing markedly dilated congested CV with detached lining (black arrow) and markedly apoptotic hepatocytes (blue arrow) with moderate micro-vesicular steatosis in peri-venular area (red arrow). F100, R100, and R300: Liver showing mildly edematous portal tract (black arrow) with mildly dilated congested portal vein (PV) and average central vein (blue arrow). F300: Liver displaying mildly edematous portal tract (black arrow), moderate portal inflammatory infiltrates (blue arrow), obviously dilated congested portal vein (PV), markedly dilated congested central vein (CV), and scattered apoptotic hepatocytes in a peri-portal area (yellow arrow). FIR100: Liver showing mildly edematous portal tract (black arrow), mildly dilated congested PV, mildly dilated CV, and large areas of necrosis (blue arrow). RIR100: Liver showing mildly edematous portal tracts (black arrow) with mildly dilated congested PV and normal central vein (blue arrow). FIR300: Liver specifying markedly edematous portal tract (black arrow) with markedly dilated congested PV and average CV with detached lining (blue arrow). RIR300: Liver specifying mildly edematous portal tract (black arrow), mildly dilated congested PV and markedly dilated CV (blue arrow).

Photomicrographs of liver tissues of γ-irradiated rats showed marked edematous portal tracts, portal inflammatory infiltrate, markedly dilated congested portal veins, markedly dilated congested central veins with detached lining, moderate apoptosis, and moderate micro-vesicular steatosis of hepatocytes in peri-portal and peri-venular areas with small areas of necrosis. The control liver showed average portal tracts, portal veins, bile ducts, hepatocytes in the peri-portal area and central veins with average hepatocytes arranged in single-cell cords with average intervening blood sinusoids (Figure 5).

Rats injected with *W. filifera* or *W. robusta* alone (F100, R100, or R300) showed mildly edematous portal tracts, mild portal inflammatory infiltrate, mild dilated congested portal veins, average central veins, average hepatocytes in peri-portal and peri-venular areas, confirming their relative safety. However, rats receiving F300 only showed marked edematous portal tracts, moderate portal inflammatory infiltrate, markedly dilated congested portal veins with detached apoptotic hepatocytes in peri-portal area, and distinctly dilated congested central veins with average hepatocytes in peri-venular area: These observations may reflect that EWR is safer than EWF, and that is in accordance with the LD_50_ study and biochemical assessments. However, the irradiated rats treated by FIR100, RIR100, or RIR300 showed mildly edematous portal tracts, mildly dilated congested portal veins, evidently dilated congested central veins with detached lining, and scattered apoptotic hepatocytes more in the peri-venular area, reflecting a modulatory effect compared with the irradiated group reflecting a hepatoprotective effect. In contrast, rat liver injected with EWF at dose 300 mg/kg displayed evidently edematous portal tracts, markedly dilated congested portal veins, markedly dilated congested central veins, and mildly apoptotic hepatocytes more in the peri-venular area. Thus, the FIR300 group did not show an obvious radio-modulatory effect.

In brief, histopathological impairments of the hepatocytes owing to gamma irradiation could be due to the increase in lipid peroxidation, which damaged the cell membrane and consequently produced inflammation and apoptosis [34,35]. Hence, the hepatoprotective effect of the extracts may be ascribed to their antioxidant and free radical scavenging properties. The results illustrated in Table 5 indicated that there was a recovery from liver damage caused by gamma irradiation in FIR100, RIR100, or RIR300 groups. Meanwhile, the EWR exerted more promising effects due to obvious inhibition of inflammation, apoptosis, and necrosis in comparison to EWF. These extracts succeeded to counteract oxidative stress induced by gamma irradiation in the liver tissues, due to their high phenolic and flavonoid contents. Moreover, assuming that the ethanolic extracts of EWR are more effective as liver protectors greatly correlates with their effect on all estimated blood parameters related to liver protection as well as genetic examination. This effect could be further confirmed through immunohistochemical examination.

### 2.7. Immunohistochemical Examination

Reports on the role of the cellular immune response in the genesis of histopathological alterations observed in liver toxicity resulting from gamma radiation are scarce. The current study is expected to provide a better understanding of the genesis of hepatic injury and the interaction with the immune response in an attempt to detect the pathogenesis of the induced liver damage. In this aspect, immunohistochemical examination of TNF-*α*, IL-6, and caspase-3 was performed.

#### 2.7.1. Effect on Hepatic TNF-*α*

**Figure 6:**
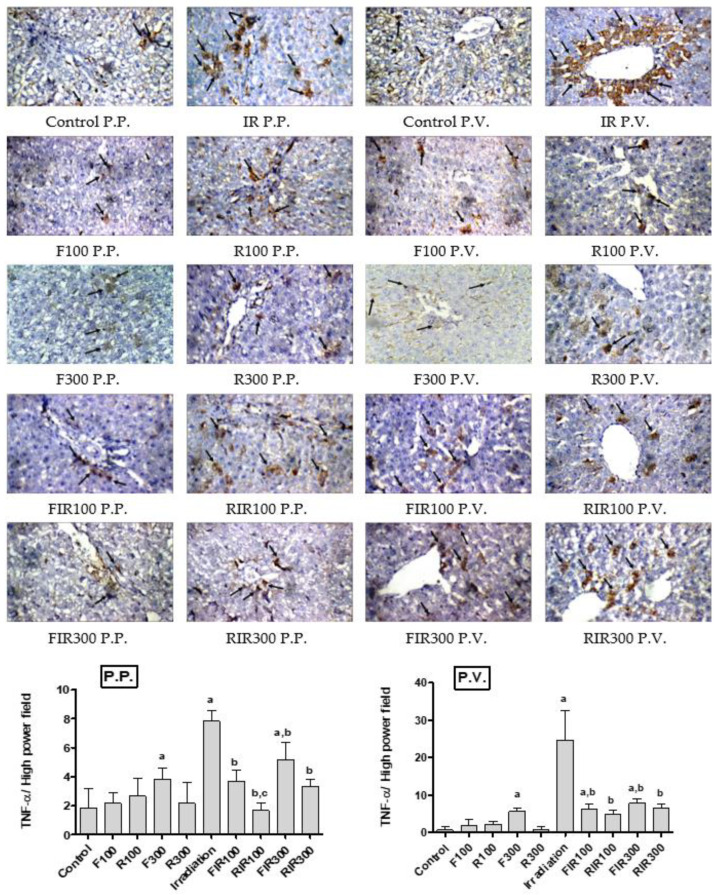
Immunohistochemical photographs of TNF-*α* expression in hepatic peri-portal (P.P.) and peri-venular (P.V.) areas in irradiated rats treated with *W. filifera* and *W. robusta* leaves ethanolic extracts (×400). Sections were taken from livers (P.P. and P.V.) of control rats showing very low expression. Sections taken from livers (P.P. and P.V.) of irradiated rats shows extensive cytoplasmic expression (brown color). Sections taken from livers (P.P. and P.V.) of irradiated rats treated with *W. filifera* or *W. robusta* showing moderate to limited expression (brown color). Data expressed as mean ± SD (*n* = 6), significance was at *p* ≤ 0.05 by means of one-way ANOVA followed by Tukey–Kramer as a post hoc-test. ^a^ Significantly different as of control group. ^b^ Significantly different as of irradiated group. ^c^ Significantly different as of FIR100.

Among all the known physiological inducers of apoptosis in cells, the tumor necrosis factor (TNF) is perhaps the most potent and well-studied one. TNF activates both cell-survival and cell-death mechanisms simultaneously [44]. In fact, TNF-*α* is a pro-inflammatory cytokine that activates NF-ĸB and increases the production of acute phase protein (CRP, C-reactive protein) in hepatocytes that in time results in the inflammatory process which increases ROS production, thus becoming an apoptotic agent [45]. Therefore, it was suggested that following liver irradiation, a sequence of events takes place, leading to disturbance of hepatocytes, cytokine release including TNF-*α* by liver macrophages, and finally hepatocellular death. Therefore, anti-TNF-*α* therapy may allow protection against radiation-induced TNF-*α* mediated cellular damage [46]. Photomicrographs of immunohistochemical staining of TNF-*α* of liver sections obtained from normal, non-irradiated-treated, and irradiated-treated animals are represented in Figure 6.

The results showed a mean of 9 positive cells in the peri-portal area (P.P.) and 29 positive cells in peri-venular area (P.V.) of the irradiated group, with high significant immunoreactivity comparing to the control group (only 3 positive cytoplasmic stains in both P.P. and P.V.), while all extract-treated groups (non-irradiated) showed no significant change in the immunohistochemical staining of TNF-*α* compared to the control group (limited immunoreactivity), confirming their safety, except treatment with F300, which showed significant immunoreactivity in both P.P. and P.V. areas. Treatment of irradiated rats with *W. filifera* or *W. robusta* extracts showed a significant decrease in TNF-*α* expression in the P.P. and P.V. areas compared with the irradiated group (moderate immunoreactivity); however, EWR showed relative lower immunoreactivity than EWF. Tested extracts may exert their hepatoprotective actions via suppressing STING as previously shown in this study and pro-inflammatory mediators including TNF-*α*. Consequently, they inhibit ROS and TNF-*α* mediated NF-κB activation and mitigate inflammation and cellular damages induced by ionizing radiation.

#### 2.7.2. Effect on Hepatic IL-6

**Figure 7:**
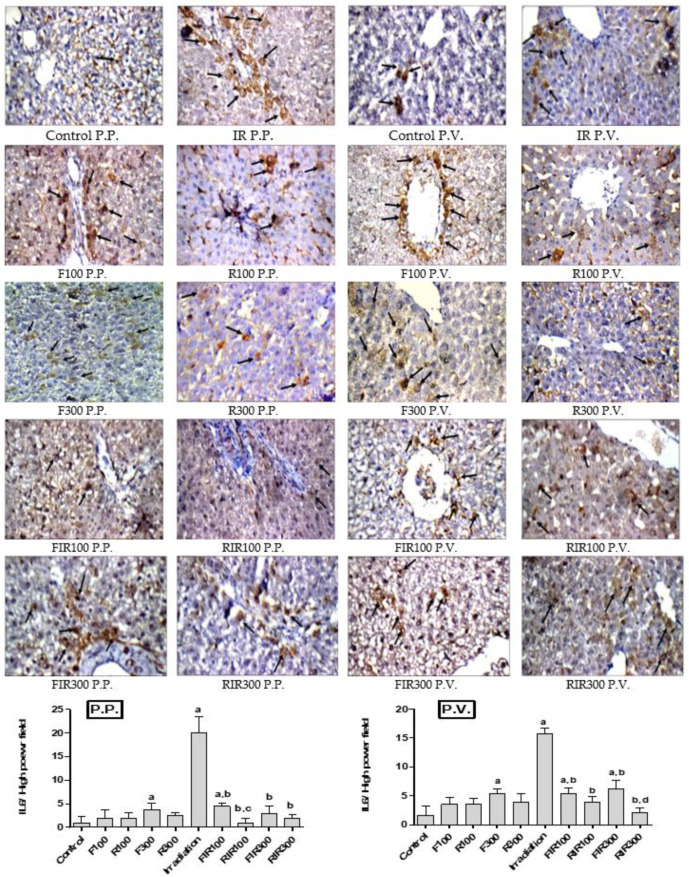
Immunohistochemical photographs of IL-6 expression in hepatic peri-portal (P.P.) and peri-venular (P.V.) areas in irradiated rats treated with *W. filifera* and *W. robusta* leaves ethanolic extract (×400). Sections were taken from livers (P.P. and P.V.) of control rats showing rare expression. Sections taken from livers (P.P. and P.V.) of irradiated rats show extensive cytoplasmic expression (brown color). Sections taken from livers (P.P. and P.V.) of irradiated rats treated with *W. filifera* or *W. robusta* show medium to limited expression (brown color). Data conveyed as mean ± SD (*n* = 6), significance was at *p* ≤ 0.05 by means of one-way ANOVA followed by Tukey–Kramer as a post hoc-test. ^a^ Significantly different as of control group. ^b^ Significantly different as of irradiated group. ^c^ Significantly different as of FIR100 group. ^d^ Significantly different as of FIR300 group.

IL-6 is an inflammatory cytokine generated in response to infections and tissue injuries, which stimulates a range of signaling pathways. It is also known to be upregulated in the liver after γ-irradiation [47], as IL-6 issues a warning signal in tissue damage, released from damaged or dying cells in non-infectious inflammations such as radiation [48]. On the other hand, IL-6 reduces the production of fibronectin, albumin, and transferrin [43]. Thus, it leads to a serious complication of several chronic inflammatory diseases [48]. Photomicrographs of immunohistochemical staining of IL-6 of liver sections obtained from normal, non-irradiated-treated, and irradiated-treated animals are represented in Figure 7.

The immunohistochemical detection of the cytoplasmic stain of hepatic IL-6 revealed no significant changes in rats injected with the two tested extracts without radiation exposure confirming their safety, except rats treated with F300 showed relative moderate immuno-reactivity, while the irradiated rats showed drastic elevation in IL-6 expression when compared with the control group in peri-portal and peri-venular areas (positive cells number; 20 and 16, in irradiated group vs 1 and 4 in control group, respectively in P.P. and P.V. areas). The IL-6 was slightly expressed in the FIR100 group (in the P.P. and P.V. areas) and FIR300 (P.V. area) compared with the control group, but RIR100 and RIR300 did not show any significant changes. Reversely, in comparison with the irradiated group, the gamma radiated-rats treated with the two extracts revealed a significant decline in IL-6 stained cells. Thus, EWF showed only a partial decrease in the recorded expression level. Therefore, treatment of irradiated rats with the tested extracts may improve γ-irradiation-induced hepatic injury and inflammation by diminishing NF-kB signaling and consequently decreasing the IL-6 level. The most pronounced effect appeared in the irradiated group treated with *W. robusta* (RIR100 in P.P. and RIR300 in P.V. area) compared to irradiated rats treated with *W. filifera* at the same dose.

#### 2.7.3. Effect on Hepatic Caspase-3

**Figure 8:**
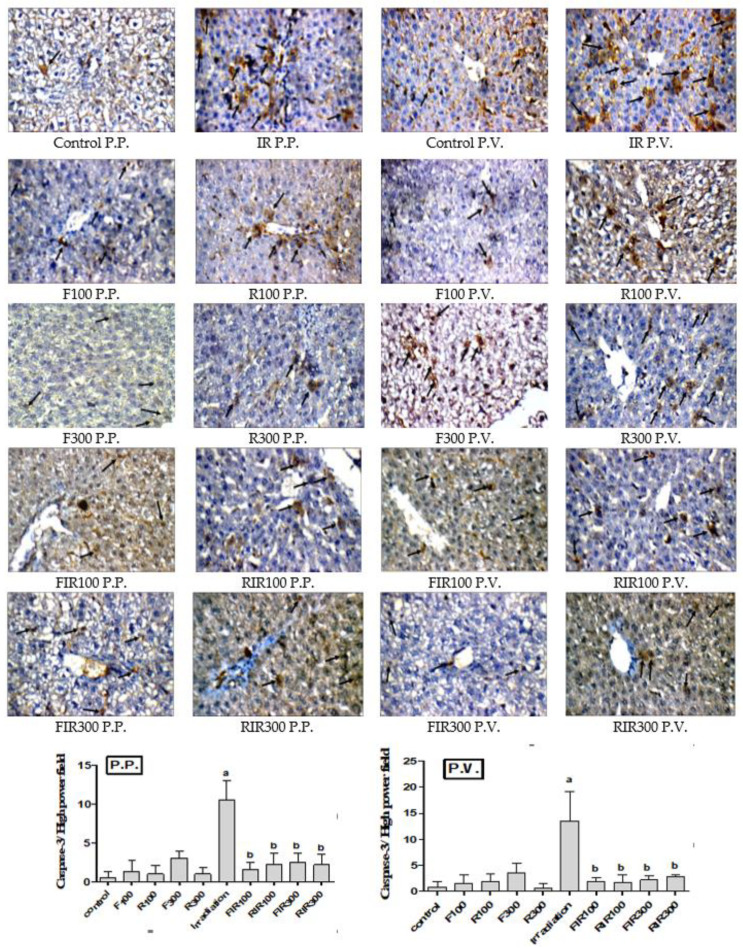
Immunohistochemical photographs of caspase-3 expression in hepatic peri-portal (P.P.) and peri-venular (P.V.) (black arrows) areas in irradiated rats treated with *W. filifera* and *W. robusta* leaves ethanolic extracts (×400). Sections taken from livers (P.P. and P.V.) of control rats showing minimal expression. Sections taken from livers (P.P. and P.V.) of irradiated rats shows extensive cytoplasmic expression (brown color). Sections taken from livers (P.P. and P.V.) of irradiated rats treated with *W. filifera* or *W. robusta* showing medium to limited expression (brown color). Data conveyed as mean ± SD (*n* = 6), significance was at *p* ≤ 0.05 by means of one-way ANOVA followed by Tukey–Kramer as a post hoc-test. ^a^ Significantly different as of control group. ^b^ Significantly different as of irradiated group.

Apoptosis (programmed cell death) is one of the best-characterized phenomena in cellular and molecular biology, and its deregulation leads to several human diseases. Caspase activation plays an essential role in the execution of apoptosis. A variety of intrinsic and extrinsic stressors are capable of initiating caspase activation [49]. In addition, caspase-3 is activated by the DNA-damage, which lead to apoptosis or inflammation [50]. Radiation seems to be able to induce an apoptotic program through ROS generation which can trigger the mitochondrial pathway to release caspase-activating-factors. Therefore, oxidative stress may play a direct role in radiation-induced apoptosis [51]. Photomicrographs of immunohistochemical staining of caspase-3 of liver sections obtained from normal, non-irradiated-treated, and irradiated-treated animals are represented in Figure 8.

Immunohistochemical staining revealed minimal caspase-3 expression in the liver sections from the control (1 and 2 positive cells in P.P. and P.V., respectively), and *W. filifera-* or *W. robusta*-only treated rats (P.P. and P.V. areas). However, the irradiated group in P.P. (11 positive cells) and P.V. (15 positive cells) areas showed significantly raised expression of caspase-3, in comparison with the control. Overexpression of caspase-3 following γ-irradiation may be due to excessive ROS generation that increases IL-6 and TNF-α, leading to cell apoptosis. Additionally, the increase in caspase-3 could attribute to STING activation by γ-irradiation that affected cell apoptosis through activation of BCL_2_-associated protein (BAX), depending on caspase-3 and -9 [37]

Compared with irradiated group, liver sections of irradiated rats post-treated with *W. filifera* or *W. robusta* showed a marked reduction in caspase-3 expression, both in P.P. and P.V. areas. These results suggested that supplementation of the tested extracts to irradiated rats downregulated caspase-3 expression (decrease the expression) and hence mitigated the antioxidant and anti-inflammatory status in the hepatic tissues. These data are compatible with the STING results for EWR, as it was recorded to be favored in reversing the harm effect of γ-radiation on liver biomarkers.

## 3. Materials and Methods

### 3.1. Plant Material

Fresh leaves of *W. filifera* H. Wendl. and *W. robusta* H. Wendl. were collected during the summer season (August–September 2018) from palms grown at the Zoological Garden (Giza, Egypt). Voucher specimens (encoded 2018.09.30 I and 2018.09.30 II) were deposited in the Herbarium of the Pharmacognosy Department, Faculty of Pharmacy, Cairo University.

### 3.2. Extraction and Samples Preparation

Air-dried, powdered leaves of the selected species (2.5 kg, each) were extracted with petroleum ether (b.r. 40–60 °C, 5 × 6 L) followed by ethanol (90%, 10 × 6 L). The extracts were evaporated under reduced pressure in a rotary evaporator (Büchi, Switzerland). The dried ethanolic extracts of *W. filifera* and *W. robusta* (encoded EWF and EWR) were weighed and their percentage yields were calculated.

### 3.3. Chemical Examination

#### 3.3.1. Proximate Composition and Phytochemical Screening

Proximate analysis of the leaves of *W. filifera* and *W. robusta* was performed by adopting the method of the Association of Official Analytical Chemists [52]. The leaves were herein subjected to determination of pharmacopoeial standards (total ash, acid-insoluble ash, water-soluble ash, crude fiber, and moisture contents) and macronutrients (fats, proteins, and carbohydrates). Experiments were carried out in triplicates. All recorded values are averages of three determinations, and expressed as percentage (*w*/*w*) with respect to the air-dried material. The air-dried, powdered leaves of the two species were subjected to preliminary phytochemical screening for the presence of different types of plant metabolites. Chemical tests were performed according to published procedures, and included the detection of the following: Steam volatile substances, oxidase enzyme, and cardiac glycosides [53]; crystalline sublimate [54]; carbohydrates and/or glycosides, tannins, saponins, sterols and/or triterpenes, alkaloids and anthraquinones [55], and flavonoids [56].

#### 3.3.2. Determination of Total Phenolic and Flavonoid Contents (TPC and TFC)

The ethanolic (90%) extracts of the tested leaves were chosen for the current work based on their aforementioned favorable response to phenolic and flavonoid assays [56]. Total phenolic and flavonoid contents (TPC and TFC) were determined colorimetrically utilizing the Folin–Ciocalteu [57] and AlCl_3_ [58] assays, respectively. The absorbance of the color produced was measured at 765 nm for TPC and 415 nm for TFC on a Shimadzu double-beam spectrophotometer (UV-1650, Shimadzu, Japan). The standard for TPC was gallic acid and quercetin for TFC. Total phenolic content was expressed as μg gallic acid equivalent (GAE)/mg dried extract, and that of flavonoids as μg quercetin equivalent (QE)/mg dried extract.

### 3.4. In Vitro Evaluation of Antioxidant Potential

FRAP and ABTS radical scavenging assays were performed on the ethanolic (90%) extracts, by means of BioVision’s FRAP Assay Kit (Theo Bhavan Rd, Mavelikara, Kerala 690102, India) and ZenBio ABTS Antioxidant Assay Kit (Research Triangle Park, NC 27709, USA), in accordance with previously described methods [24,25,26] for FRAP and [21,22,23] for ABTS. The color absorbance produced was recorded on a Robonik P2000 ELISA reader at 594 nm for FRAP, and at 405 nm for ABTS. Ascorbic acid was used as a standard antioxidant in both methods.

### 3.5. Isolation and Identification of Phenolic Components of EWR

An aliquot (180 g) of dried EWR was suspended in distilled water followed by fractionation with dichloromethane, ethyl acetate, and *n*-butanol saturated with water in succession. The dried ethyl acetate extract (30 g) was chromatographed using VLC (vacuum liquid chromatography) on a Silica gel H 60 (Merck, Darmstadt, Germany) column (500 g). Gradient elution was applied with methylene chloride, ethyl acetate, and methanol. The individual extractives were subjected to TLC on pre-coated silica gel 60 F 254 plates (Fluka, Sigma–Aldrich Chemicals, Munich, Germany); the spots were visualized by exposure to UV_365nm_ and spraying with AlCl_3_ and/or FeCl_3_ reagents. Further purification of the selected fractions was carried out on Sephadex LH-20 (Pharmacia Fine Chemicals AB, Uppsala, Sweden) and Silica gel 60 (Sigma–Aldrich Chemicals, Munich, Germany) columns. The structures of isolated secondary metabolites were elucidated based on physicochemical and spectral data (m.p., m.m.p. co-TLC, UV, ^1^H NMR, and ^13^C NMR). The melting points were measured using an electrothermal IA9100 apparatus (Burlington, NJ, USA). ^1^H NMR and ^13^C NMR spectroscopy were recorded by a Bruker NMR-spectrometer 400 MHz (Bruker, Japan).

### 3.6. In vivo Evaluation of Antioxidant Protective Potential in γ-Irradiated Rats

#### 3.6.1. Experimental Animals and Laboratory Diet

The experimental protocol applied in the current study was approved by the Institutional Animal Care and Use Committee of Cairo University (CU-IACUC), approval code; CU III F 84 19. Adult female Wistar rats (130–150 g) and Swiss albino mice (22–25 g) were obtained from the National Centre for Radiation Research and Technology (NCRRT), Cairo, Egypt. Animals were acclimatized for one week before starting the experiment being housed in a NCRRT animal house. They were maintained under standard laboratory conditions (controlled room temperature, 26 ± 2 °C; relative humidity, 54–68%, and 12 h light/dark cycle), and with free access to a well-balanced diet (vitamin mixture, 1%; mineral mixture, 4%; corn oil, 10%; sucrose, 20%; cellulose, 0.2%; casein, 10.5%; and starch, 54.3%) and water. Squeezing, pressure, and hard handling of animals were avoided.

#### 3.6.2. Irradiation Process

Gamma-irradiation was achieved at the National Centre for Radiation Research and Technology (NCRRT), Atomic Energy Authority, Cairo, Egypt, using Canadian Gamma Cell-40 biological radiator (^137^Cesium), manufactured by the Atomic Energy of Canada Limited, Ontario, Canada. The radiation dose rate was 0.403 Gy/min at the time of exposure. The total radiation dose was 7.5 Gy as a whole body shot dose [59].

#### 3.6.3. Determination of the Median Lethal Dose (LD_50_)

Safety or acute toxicity of the tested extracts was investigated through the determination of their median lethal doses (LD_50_, dose of plant extracts causing death of 50% of the treated animals within 24 h post treatment). The lethal dose was estimated (Table 6) using the method of Chinedu et al. [60], following 3 stages (one mouse/group).

The animals were observed for 1 h post-administration (i.p.) and periodically for further 24 h. Testing should be stopped at the stage at which mortality occurred, and not proceeded to the next stage. When mortality was recorded at a dose in any stage, a confirmatory test was carried out in order to validate the mortality dose, using two mice where at least a single mouse died. LD_50_ was determined according to the following formula:

LD_50_ = [M_0_+M_1_]/2 (1)

where M_0_ = highest dose of tested extract that gave no mortality, M_1_ = lowest dose of tested extract that gave mortality.

#### 3.6.4. Experimental Design

Sixty female rats were divided into 10 groups (*n* = 6) as follows:

Group 1: Served as negative control animals. Group 2 (F100): Rats were injected with EWF (100 mg/kg b.wt., i.p.). Group 3 (R100): Rats treated with EWR (100 mg/kg b.wt., i.p.). Group 4 (F300): Rats received 300 mg/kg b.wt. (i.p.) of EWF. Group 5 (R300): Rats were given EWR (300 mg/kg b.wt., i.p.). Group 6 (Irradiation): Rats irradiated with 7.5 Gy as a shot dose and served as a positive control [59]. Group 7 (FIR100): Rats irradiated with 7.5 Gy followed by i.p. injection of EWF (100 mg/kg b.wt.). Group 8 (RIR100): Rats were exposed to 7.5 Gy γ-rays followed by i.p. injection of EWR (100 mg/kg b.wt.). Group 9 (FIR300): Rats were exposed to 7.5 Gy γ-rays followed by i.p. injection of EWF (300 mg/kg b.wt.). Group 10 (RIR300): Animals were exposed to 7.5 Gy γ-rays followed by i.p. injection of EWR (300 mg/kg b.wt.). Seven days later after irradiation, all animals were anesthetized by i.p. injection of urethane at the dose of 1.2 g/kg i.p. [61]. Blood samples were collected by heart puncture, and then rats were human-killed by cervical dislocation. The blood samples were centrifuged at 4000 rpm for 10 min. The separated sera were used for measurement of alanine aminotransferase (ALT), albumin, total cholesterol (TC), triglyceride (TG), reactive oxygen species (ROS) and malondialdehyde (MDA), while livers were removed, cleaned with normal saline solution, dried, and divided into two portions. A portion was homogenized in ice-cold saline and utilized for relative gene expression ratio of STING (stimulator of interferon gene). Meanwhile, the second was fixed in 10% formalin solution for histopathological examination and immunohistochemical (TNF-*α*, IL-6 and caspase-3) analysis.

#### 3.6.5. Biochemical Assays

##### In Vivo Evaluation of Liver Biomarkers

Lipid and liver function parameters (TC, ALT, and albumin) were estimated by means of specific diagnostic kits (Biomed, Germany), triglyceride (TG) by Biodiagnostic, Egypt, in accordance with previously described methods; TC [62], TG [63], ALT [64], and albumin concentrations [65].

##### In Vivo Evaluation of Oxidative Stress Parameters

Sera of samples were used for spectrophotometric estimation of reactive oxygen species (ROS) according to Vrablic et al. [66] and lipid peroxidation (malondialdehyde; MDA formation) according to Yoshioka et al. [67].

#### 3.6.6. Genetic Profiling; RNA Extraction and RT-PCR Analysis of the Stimulator of Interferon Gene (STING) in Liver

Total RNA was isolated from hepatic tissue homogenate by means of RNeasy Purification Reagent (Qiagen, Valencia, CA, USA) in accordance with the manufacturer’s protocol. Extracted RNA was calculated spectrophotometrically at 260 nm. Reverse transcription was performed on 5 μg RNA from each tested sample utilizing MMuLV reverse transcriptase in a 50-μL reaction volume. The reverse transcription mixtures were used for amplification of fragments specific for STING by PCR using the primer pairs: F: 5′GGACAGTCGCTGTGACCGAGGTT3′, R: 5′GCTTGTTGGGCCAGTCCTGATG3′ (gene bank accession number 025142159.1.). The real time PCR was performed by the QuantiTect SYBR green PCR Kit (Qiagen, Germany) in accordance with the manufacturer’s instructions, by Applied Biosystems 7500 Instrument, USA. The PCR reaction mix was carried out in a total volume of 25 μL, containing 2× QuantiTect SYBR green PCR master mix, 20 pmol/μL specific primer. Records were evaluated by the ABI Prism sequence detection system software and quantified by the v1·7 Sequence Detection Software from PE Biosystems (Foster City, CA, USA). The relative STING gene expression was evaluated by the comparative threshold cycle method. All values were normalized to the *β*-actin genes (F: 5′AGGCATCCTCACCCTGAAGTA3′ R: 5′CACACG-CAGCTCATTGTAGA3′) (gene bank accession number NM 001009945.1) as an invariant endogenous control. The relative quantification was then calculated by the fold changes, ΔCT = ΔCT_samples_ − ΔCT_control_.

#### 3.6.7. Histopathological Examination

Paraffin sections (4 µm thick) of buffered formalin-fixed liver were prepared directly after scarification (5 samples of each group). Gamma radiation-induced inflammation, apoptosis, and necrosis was estimated according to the morphological changes in liver sections stained with hematoxylin and eosin (H and E) [68]. Histopathological processing and assessment of specimens were performed by the aid of a skilled histologist, blinded to the studied samples identity to avoid any bias.

#### 3.6.8. Immunohistochemical Analysis

Formalin-fixed, paraffin-embedded, 4-μm-thick sections of liver tissues were used for immunohistochemical staining. Immunohistochemical staining of TNF-*α* (rabbit polyclonal AB purchased from Santa Cruz Biotechnology, Santa Cruz, CA diluted at 1:100, in phosphate buffered saline (PBS)), of IL-6 (rabbit polyclonal AB purchased from Santa Cruz Biotechnology, Santa Cruz, CA, USA), and of caspase-3 (rabbit polyclonal AB-3 purchased from Lab Vision Co., Fremont, CA, USA) were carried out and scored in two different areas of the hepatic tissue (peri-portal and peri-venular areas), in accordance with Kasprzak et al. [69]. Reactivity was mainly the number of cytoplasmic nuclear staining per high power field of the specimens.

### 3.7. Molecular Docking of the Isolated Compounds in the Stimulator of Interferon Gene (STING) Active Site

The 2D structures of the 4 compounds were minimized from the energy point of view to obtain valid 3D structures required for docking using Baloon software. Each 3D compound structure was then converted to the PDBQT file format required for the docking stage using a python routine. The active site of the receptor was defined using the MGTOOLS software within the Vina package [70]. The dimensions of the grid box were defined to 40 × 40 × 40 in the three dimensions of space and centered around the point whose three-dimensional coordinates are center_*x* = 63.444, center_*y* = −14.297, center_*z* = 78.297. The docking of the compounds at the active site of the receptor was carried out using the Vina software. All Vina settings were kept at their default values. The 2D structures of the 4 compounds naringenin, kaempferol, quercetin, and gallic acid were obtained from the PubChem database. The 3D structure of the STING receptor (PDB code: 6DXL) was extracted from the PDB dataBank database.

### 3.8. Statistical Analysis

The data were expressed as mean ± standard deviation (SD) (*n* = 6). All in vivo results were analyzed by one-way ANOVA followed by Tukey–Kramer as a post hoc-test, using GraphPad Prism 5 software (San Diego, CA, USA). Differences were considered significant at *p* < 0.05.

## 4. Conclusions

The current results verified that both *Washingtonia filifera* and *W. robusta* leaf extracts significantly alleviate gamma radiation-induced oxidative stress and subsequent liver impairment. Moreover, this study could be considered as one of the earliest elucidations of the mechanism of the radioprotective potential of *W. filifera* and *W. robusta* leaf extracts. This effect was found to be mediated by the inhibition of oxidative stress, and consequently decreasing DNA damage that blocks STING activation, and hence hinders inflammation (TNF-α, IL-6) and apoptosis (caspase-3) induction. The results of the histopathological examination of liver tissues indicated coincidence with those noted by the biochemical examination. In addition, the ethanolic extract of the defatted leaves of *W. robusta* could be favored as a pharmaceutical candidate rather than that of *W. filifera*, taking into consideration its relatively higher yield, greater phenolic contents, and more pronounced in vitro and in vivo bioactivities. It was also noticeable that this extract produces the same effect in all investigated in vivo parameters whether given at high (300 mg/kg.b.wt.) or low doses (100 mg/kg.b.wt.). Consequently, the lower dose can provide more relevant bioactivity by avoiding the possible side effects of repeated or long-term administration of the high dose. Finally, the protective capacity of *W. robusta* extract against gamma radiation-induced hepatotoxicity was obviously associated with its phenolic components through their ability to interact with STING, as indicated in the in-silico studies. These findings support the fact that some herbal products have better prospects as radioprotectors because they are promising sources of potential natural antioxidants. However, further clinical trials are necessary to enable their implementation in pharmaceutical formulations.

## Figures and Tables

**Table 1:** Physical examination of the extracts of the leaves of *Washingtonia filifera* and *W. robusta*.

Yield and Physical Characters	Petroleum Ether		Ethanol 90%		Methylene Chloride		Ethyl Acetate		*n*-Butanol	
	WF	WR	WF	WR	WF	WR	WF	WR	WF	WR
Yield (g) (%)	145 (5.8%)	175 (7%)	170 (6.8%)	210 (8.4%)	33 (1.32%)	50 (2%)	45 (1.8%)	60 (2.4%)	22 (0.8%)	28 (1.12%)
Condition	Solid		Semi-solid		Solid		Solid		Solid	
Color	Dark green		Dark brown		Green		Reddish brown		Reddish brown	
Taste	Waxy		N.C.		N.C.		N.C.		N.C.	
Odor	Odorless		Odorless		Odorless		Odorless		Odorless	

Yield (g) (% Yield, g/100g dried powder); (N.C.) Not characteristic; (WF) *W. filifera*; (WR) *W. robusta*.

**Table 2:** Antioxidant activity of the leaves of *W. filifera* and *W. robusta*.

Assays	Extracts		Ascorbic Acid
	*W. filifera*	*W. robusta*	
ABTS (μM TE/g)	223.8 ± 7.79	239.5 ± 6.21	278.9 ± 14.1
FRAP (mM Ferrous Equivalents)	75.79 ± 3.2	81.58 ± 3.5	123.95 ± 3.29

TE/g: Trolox equivalents per gram sample. Values are averages of three determinations ± SD.

**Table 3:** Effect of *W. filifera* and *W. robusta* leaves ethanolic extracts on malondialdehyde (MDA) and reactive oxygen species (ROS) levels in serum of irradiated rats.

Group	Parameter	
	MDA (nmol/L)	ROS (NBT/mL)
Control	41.1 ± 4.523	81.5 ± 8.083
F100	34.4 ± 6.4	103 ± 19.10
R100	34.08 ± 2	75.76 ± 17.21
F300	36.31 ± 5.7	126.4 ± 15.23 ^a^
R300	39.48 ± 4.4	102.7 ± 22.4
Irradiation (7.5 Gy)	57.83 ± 9.6 ^a^	143.1 ± 10.73 ^a^
FIR100	42.20 ± 6.1 ^b^	133.8 ± 16.36 ^a^
RIR100	40.79 ± 8.2 ^b^	83.20 ± 10.11 ^b,c^
FIR300	41.44 ± 4.8 ^b^	117.6 ± 11.57 ^b^
RIR300	40.56 ± 5.3 ^b^	112.6 ± 14.7 ^b^

Data expressed as mean ± SD, significance was at *p* ≤ 0.05 by means of one-way ANOVA followed by Tukey–Kramer as a post hoc-test. ^a^ Significantly different as of control, ^b^ significantly different as of irradiated group, ^c^ significantly different as of FIR100.

**Table 4:** Effect of *W. filifera* and *W. robusta* leaves ethanolic extracts on ALT, albumin, cholesterol and triglyceride levels in serum of irradiated rats.

Group	Parameter			
	ALT (U/L)	Albumin (g/dL)	Cholesterol (mg/dL)	Triglyceride (mg/dL)
Control	10.3 ± 1.3	4.0 ± 0.87	42.7 ± 1.5	71.2 ± 7.4
F100	10.7 ± 2.3	3.0 ± 0.31 ^a^	55.4 ± 1.44	61 ± 8.4
R100	12.8 ± 0.9	4.3 ± 0.48	48.8 ± 11.58	72.7 ± 10.4
F300	10.2 ± 2.1	3.2 ± 0.13 ^a^	45.9 ± 10.10	71.1 ± 2.8
R300	8.4 ± 0.6	3.9 ± 0.13	42.9 ± 8.28	61.7 ± 3.9
Irradiation (7.5 Gy)	18.0 ± 2.3 ^a^	2.7 ± 0.44 ^a^	102.9 ± 6.14 ^a^	107.8 ± 10.1 ^a^
FIR100	17.1 ± 2.6 ^a^	3.3 ± 0.44 ^a^	56.2 ± 5.41 ^b^	80.8 ± 8.1 ^b^
RIR100	9.3 ± 1.2 ^b,c^	4.2 ± 0.15 ^b^	34.1 ± 6.83 ^b,c^	80.3 ± 7.8 ^b^
FIR300	22.4 ± 1.1 ^a,b^	2.6 ± 0.36 ^a^	50.8 ± 4.13 ^b^	80.7 ± 7.9 ^b^
RIR300	6.4 ± 0.7 ^b,d^	4.0 ± 0.37 ^b,d^	31.5 ± 4.13 ^b,d^	75.5 ± 4.4 ^b^

Data were expressed as mean ± SD, significance was at *p* ≤ 0.05 by means of one-way ANOVA followed by Tukey–Kramer as a post hoc-test. ^a^ Significantly different from control, ^b^ significantly different as of irradiated group, ^c^ significantly different as of FIR100, ^d^ Significantly different from FIR300.

**Table 5:** Histopathological scoring of liver in normal and irradiated rats treated with *W. filifera* and *W. robusta* leaves ethanolic extracts.

Group	P.T.	P.V.	P.P. Hepatocytes	C.V.	Blood Sinusoids	P.V. Hepatocytes	Intra-lobular Inflammatory Infiltrate	Necrosis
Control	0%	20%: +	0%	20%: +	0%	0%	0%	0%
F100	25%: + 75%: 0	50%: + 50%: 0	0%	50%: + 50%: 0	0%	0%	0%	0%
R100	50%: + 50%: 0	75%: + 25%: 0	0%	25%: + 75%: 0	0%	0%	0%	0%
F300	25%: + 25%: ++	50%: + 25%: ++	25%: + 75%: 0	40%: + 25%: ++	0%	25%: + 75%: 0	25%:+ 75%: 0	0%
R300	40%: + 60%: 0	60%: + 40%: 0	20%: + 80%: 0	20%: + 20%: ++	0%	0%	0%	0%
IR	50%: + 25%: ++	25%: + 75%: ++	75%: + 25%: ++	50%: + 50%: ++	50%: + 50%: 0	75%: + 25%: ++	25%:+ 75%: 0	75%: + 25%: 0
FIR100	66.6%: + 33.3%: 0	100%: +	66.6%: + 33.3%: 0	66.6%: + 33.3%: 0	66.6%: + 33.3%: 0	33.3%: + 33.3%:++ 33.3%: 0	0%	0%
RIR100	75%: + 25%: 0	50%: + 25%: ++	75%: + 25%: ++	75%: + 25%: ++	25%: + 75%: 0	50%: + 25%: ++	0%	0%
FIR300	25%: + 25%: ++	25%: + 25%: ++	50%: + 50%: 0	25%: 0 75%: ++	0%	75%: + 25%: 0	50%:+ 50%: 0	25%: + 25%: ++ 50%: 0
RIR300	75%: + 25%: 0	50%: + 50%: 0	25%: + 75%: 0	25%: + 75%: ++	0%	25%: + 75%: 0	0%	0%

Portal tract (P.T.): 0: Average +: Mild edema/inflammation ++: Marked edema/inflammation; Portal vein (P.V.): 0: Average +: Mildly dilated/congested ++: Markedly dilated/congested; Peri-portal (P.P.): 0: Average +: Scattered/mild apoptosis ++: Moderate marked apoptosis; Central vein (C.V.): 0: Average +: Mildly dilated/congested ++: Markedly dilated/congested; Blood sinusoids: 0: Average +: Mildly dilated/congested ++: Markedly dilated/congested.; Peri-venular (P.V.): 0: Average +: Scattered apoptosis ++: Mild/moderate apoptosis; Inflammatory infiltrate: 0: No +: Mild ++: Moderate/marked; Necrosis: 0: No +: Small areas ++: Large areas.

**Table 6:** Schedule for determination of lethal doses of the tested extracts.

Stage	Recommended Doses (mg/kg b.wt.)			
	Group 1	Group 2	Group 3	Group 4
1	50	200	400	800
2	1000	1500	2000	-
3	3000	4000	5000	-

## Abbreviations

ABTS	2:2-azinobis (3-ethylbenzothiazoline-6-sulfonic acid)
ALT	alanine aminotransferase
DPPH	2,2-diphenyl-1-picrylhydrazyl
EWF	defatted ethanolic extract of *W. filifera*
EWR	defatted ethanolic extract of *W. robusta*
FRAP	ferric reducing antioxidant power
GAE	gallic acid equivalent
H and E	hematoxylin and eosin
IL-6	interleukin-6
LD_50_	median lethal dose
MDA	malondialdehyde
NF-ĸB	nuclear factor kappa B
P.P.	peri-portal area
P.V.	peri-venular area
PBS	phosphate-buffered saline
QE	quercetin equivalent
ROS	reactive oxygen species
STING	stimulator of interferon gene
TC	total cholesterol
TFC	total flavonoid content
TNF-*α*	tumor necrosis factor alpha
TPC	total phenolic content
