# Correction: Selim et al. Impact of *Washingtonia robusta* Leaves on Gamma Irradiation-Induced Hepatotoxicity in Rats and Correlation with STING Pathway and Phenolic Composition. *Pharmaceuticals* 2020, *13*, 320

**DOI:** 10.3390/ph17111481

**Published:** 2024-11-05

**Authors:** Nabil M. Selim, Seham S. El-Hawary, Soheir M. El Zalabani, Rehab Nabil Shamma, Nariman El Sayed Mahdy, Noheir H. Sherif, Hanan A. Fahmy, Mai H. Mekkawy, Abdelaziz Yasri, Mansour Sobeh

**Affiliations:** 1 Department of Pharmacognosy, Faculty of Pharmacy, Cairo University, Cairo 12613, Egypt; seham.elhawary@yahoo.com (S.S.E.-H.); selzalabani@gmail.com (S.M.E.Z.); narimanmahdy@yahoo.com (N.E.S.M.); 2 Department of Pharmaceutics and Industrial Pharmacy, Faculty of Pharmacy, Cairo University, Cairo 12613, Egypt; rehab.shamma@pharma.cu.edu.eg; 3 Pharmacognosy Department, Faculty of Pharmacy, Nahda University, Beni-Suef 62513, Egypt; noheir_1@hotmail.com; 4 Drug Radiation Research Department, National Centre for Radiation Research and Technology, Egyptian Atomic Energy Authority, P.O. Box: 29 Nasr City, Cairo 11865, Egypt; fahmy.hanan@yahoo.com (H.A.F.); maimekkawy@hotmail.com (M.H.M.); 5 AgroBioSciences Research Division, Mohammed VI Polytechnic University, Lot 660–Hay MoulayRachid, Ben-Guerir 43150, Morocco; aziz.yasri@um6p.ma

## Error in Figure

In the original publication [1], there was a mistake in Figures 7 and 8 as published. “R300 P.V./TNF alpha” at Figure 6 was mistakenly used again for “F300 P.V./IL-6” at Figure 7; “F100 P.P./TNF alpha” at Figure 6 was mistakenly used again for “F100 P.V./caspase-3” at Figure 8; “FIR300 P.V./IL6” at Figure 7 was mistakenly used gain for “F300 P.V./caspase-3” at Figure 8. The corrected Figure 7 and Figure 8 appear below. The authors state that the scientific conclusions are unaffected. This correction was approved by the Academic Editor. The original publication has also been updated.

## Figures and Tables

**Figure 7:**
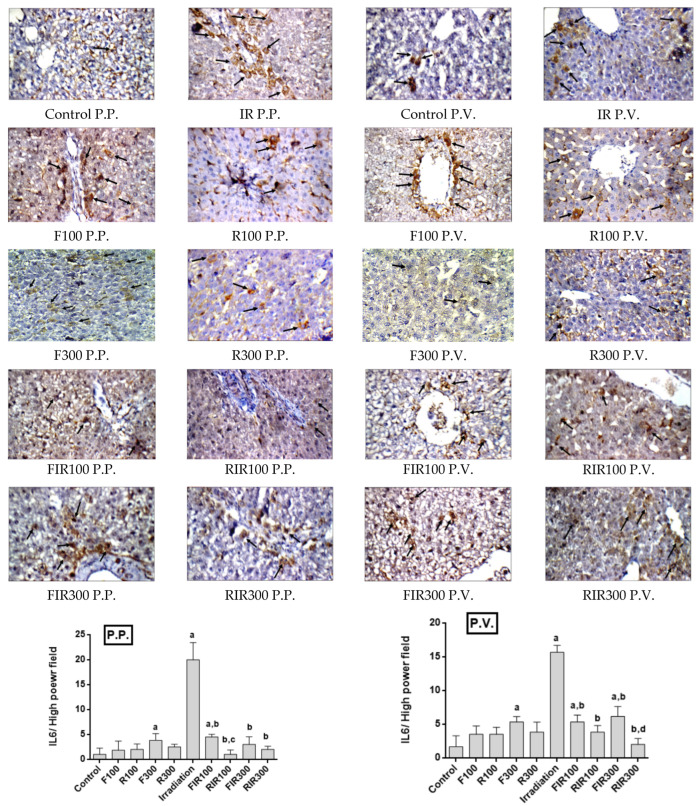
Immunohistochemical photographs of IL-6 expression in hepatic peri-portal (P.P.) and peri-venular (P.V.) areas in irradiated rats treated with *W. filifera* and *W. robusta* leaves ethanolic extract (×400). Sections were taken from livers (P.P. and P.V.) of control rats showing rare expression. Sections taken from livers (P.P. and P.V.) of irradiated rats show extensive cytoplasmic expression (brown color). Sections taken from livers (P.P. and P.V.) of irradiated rats treated with *W. filifera* or *W. robusta* show medium to limited expression (brown color). Data conveyed as mean ± SD (*n* = 6), significance was at *p* ≤ 0.05 by means of one-way ANOVA followed by Tukey–Kramer as a post hoc-test. ^a^ Significantly different as of control group. ^b^ Significantly different as of irradiated group. ^c^ Significantly different as of FIR100 group. ^d^ Significantly different as of FIR300 group.

**Figure 8:**
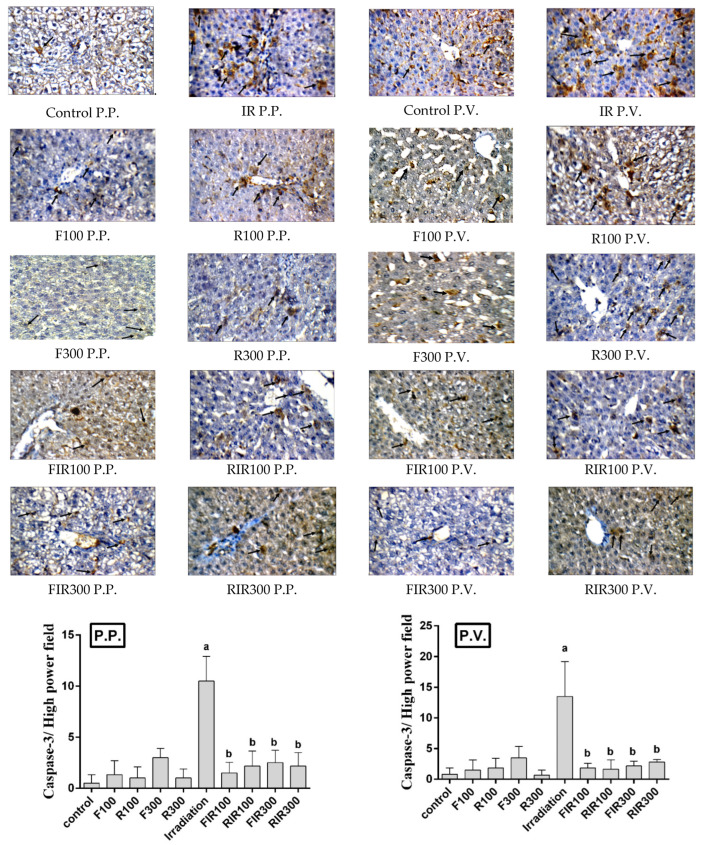
Immunohistochemical photographs of caspase-3 expression in hepatic peri-portal (P.P.) and peri-venular (P.V.) (black arrows) areas in irradiated rats treated with *W. filifera* and *W. robusta* leaves ethanolic extracts (×400). Sections taken from livers (P.P. and P.V.) of control rats showing minimal expression. Sections taken from livers (P.P. and P.V.) of irradiated rats shows extensive cytoplasmic expression (brown color). Sections taken from livers (P.P. and P.V.) of irradiated rats treated with *W. filifera* or *W. robusta* showing medium to limited expression (brown color). Data conveyed as mean ± SD (*n* = 6), significance was at *p* ≤ 0.05 by means of one-way ANOVA followed by Tukey–Kramer as a post hoc-test. ^a^ Significantly different as of control group. ^b^ Significantly different as of irradiated group.
